# Sustainable preventive integrated child health care: reflections on the importance of multidisciplinary and multisectoral stakeholder engagement

**DOI:** 10.1080/16549716.2023.2173853

**Published:** 2023-02-10

**Authors:** Olivia Biermann, Alma Nordenstam, Tonny Muwonge, Lydia Kabiri, Grace Ndeezi, Tobias Alfvén

**Affiliations:** a Department of Global Public Health, Karolinska Institutet, Solna, Sweden; b Centre of Excellence for Sustainable Health, Makerere University, Kampala, Uganda; c Centre of Excellence for Sustainable Health, Karolinska Institute, Solna, Sweden; d School of Public Health, Makerere University, Kampala, Uganda; e School of Health Sciences, Department of Nursing, Makerere University, Kampala, Uganda; f School of Medicine, Makerere University, Kampala, Uganda; g Sachs’ Children and Youth Hospital, Stockholm, Sweden

**Keywords:** Children under 5, prevention, sustainable development goals, Uganda, contextualisation, collaboration

## Abstract

Worldwide, 85% of all children who die are under the age of five. A recent scoping review examining the literature from 2000 to 2021 shows the importance of sustainable integrated preventive child health care for improving child health, enhancing the uptake of preventive child health services, and decreasing health-care costs. In 2022, we organised a stakeholder workshop in Uganda to discuss and contextualise the findings of the scoping review. The workshop took place under the umbrella of the Centre of Excellence for Sustainable Health, a virtual collaborative centre co-hosted by Makerere University in Uganda and Karolinska Institutet in Sweden. The workshop convened multidisciplinary and multisectoral stakeholders, including parents, nurses, paediatricians, nutritionists, village health team members, religious leaders, social workers, teachers, lawyers, health and climate researchers, and representatives from the police, the agricultural sector, the Ministry of Health, the World Health Organization, and other international and national non-governmental organisations, among others. We reflect on the importance of multidisciplinary and multisectoral stakeholder engagement, not only in building bridges between research and practice but also in linking sectors and connecting people for sustainable preventive integrated child health care. Though an important step, this workshop was only a first step; over time, relationships must be nurtured, multisectoral systems built and research and policy closely connected. We hope this workshop will not remain a one-off event but becomes an institutionalised effort that sparks action for sustainable preventive integrated child health care in Kampala and beyond, and sustainable health for all.

## Short communication

Worldwide, 85% of all children who die are under the age of five [1]. A recent scoping review examining the literature from 2000 to 2021 shows the importance of sustainable integrated preventive child health care for improving child health, as well as for enhancing the uptake of preventive child health services and decreasing health-care costs [2]. However, gaps between research and practice remain; many countries still deliver interventions such as vaccinations and growth monitoring as single interventions.

As part of the work of the Centre of Excellence for Sustainable Health (CESH), we organised a face-to-face stakeholder workshop in Kampala, Uganda, to discuss and contextualise the findings of the scoping review. CESH is a virtual collaborative centre co-hosted by Makerere University in Uganda and Karolinska Institutet in Sweden, aiming to develop capacity and mobilise actions to drive the agenda for sustainable health [3].

The stakeholder workshop took place on 3 May 2022, 9:00–12:00, convening 39 multidisciplinary and multisectoral stakeholders, including parents, nurses, paediatricians, nutritionists, counsellors, village health team members, religious leaders, social workers, teachers, school principals, lawyers, health and climate researchers, as well as representatives from the police, the agricultural sector, the Ministry of Health, the World Health Organization (WHO), and other international and national non-governmental organisations.

For the preparation, implementation, and follow-up of the workshop, we used a WHO checklist for policy dialogues for guidance [4]. TM, GN, and LK created an initial list of 46 potential participants, considering input from knowledgeable colleagues and other key stakeholders. All potential participants received an invitation letter for the workshop from the office of the Dean of the Makerere University School of Public Health. TM then followed up twice via mobile phone calls to confirm participation. Of the seven participants who were invited but did not attend the workshop, three were unavailable at the suggested date and four were prevented from attending at the last minute. All participants received a transportation allowance of 50,000 UGX (approximately 14 USD). After the workshop, we shared a one-page summary of the workshop and a short evaluation form with the participants via email. The anonymous evaluation included five multiple choice and three open-ended questions about the participants’ experience at the workshop, e.g. what participants liked/disliked about the workshop and how future workshops could be organised in a better way. We received feedback from 11 participants.

Here, we reflect on the importance of multidisciplinary and multi-sectoral stakeholder engagement, which remains untapped in many low- and middle-income countries [5]. Our reflections are about building bridges between research and practice, linking sectors, and connecting people for sustainable preventive integrated child health care. These reflections are based on discussions within our team, informed by participants’ feedback from the evaluation and examples from the literature:

1. **Bridging research and practice**: Applying the findings from the scoping review (and other research) requires contextualisation in a given setting. The stakeholder workshop served as a first contextualisation effort; the scoping review findings were presented, discussed, and complemented through key stakeholders’ tacit knowledge. For example, the workshop participants highlighted that the implementation of sustainable preventive integrated child health care in Kampala would require the consideration of contextual issues, such as clean water and sanitation, child marriage, teenage pregnancies, domestic violence, child labour, as well as corruption and lack of resources. The participants furthermore emphasised that the implementation of sustainable preventive integrated child health care may be challenged by several competing priorities at health facilities. The use of stakeholder dialogues to bridge the know-do-gap has also been highlighted by organisations such as the WHO, e.g. through the WHO Evidence-informed Policy Network (EVIPNet) [4,6].
2. **Linking sectors**: Sustainable preventive child health care cannot be achieved by the health sector alone; the fact that all sectors impact health makes their involvement imperative for meaningful action. The participants appreciated the group discussions with diverse stakeholders, describing the discussions as ‘extremely engaging’ and ‘very enriching’ in the evaluation. Furthermore, they stated having come to new insights on the contribution of different sectors to child health, e.g. a) informing their teaching or their community advocacy, b) clarifying roles and responsibilities, and c) improving networking, coordination, and collaboration. Several participants (e.g. parents and climate researchers) had been surprised about having been invited to the workshop but soon realised the value of contributing their views to the discussions.
  During the workshop, the stakeholders discussed multisectoral barriers and solutions. For instance, many schools lack nurses and even where nurses are available, their equipment or education may be limited to provide high-quality care. Thus, barriers regarding logistics and leadership were discussed as well as solutions related to capacity building and community training. The participants also highlighted the role of youth engagement for the successful implementation of sustainable preventive integrated child health care, not only because they make up a large portion of parents of under 5-year-olds but because their skills can be harnessed to bridge the gap in service delivery. For example, participants proposed the utilisation of students in medical fields to support immunisation campaigns and community programmes for improving child health.
3. **Connecting people**: Though concerned with the same issues, some key stakeholders had never interacted or sat around the same table before. As such, the stakeholder workshop also served as a means for building relationships and mutual trust. In the evaluation, a participant stated that stakeholders could ‘learn from each other and understand different perspectives’. The participants got to meet and interact and even formulated a joint vision for child health: a city, Kampala, where all children go to school and are fully vaccinated, and where children and families are supported, protected, and receive timely services. A previous study underlined the importance of stakeholder engagement for generating shared visions and fostering the development of coalitions that can mount organised local and regional advocacy and action for specific health topics [7]. Connecting people and building relationships and trust are paramount for the above, bridging research and practice and linking sectors.

This stakeholder workshop exemplified a multidisciplinary and multisectoral exchange effort that helped build bridges between research and practice, link sectors, and connect people for sustainable preventive integrated child health care. Though an important step, this workshop was only a first step; over time, relationships must be nurtured, multisectoral systems built, and research and policy closely connected. The WHO has furthermore emphasised the need to train key stakeholders in coordinating and structuring multisectoral work in practice, as well as to develop tools for planning, implementing, and monitoring multisectoral action [8]. We hope this workshop will not remain a one-off event but becomes an institutionalised effort that sparks action for sustainable preventive integrated child health care in Kampala and beyond, and sustainable health for all. We welcome efforts to synthesise research on the ‘how to’ of multidisciplinary and multisectoral stakeholder engagement [9] as well as new implementation research on the topic.

## Paper context

A recent scoping review showed the importance of sustainable integrated preventive child health care globally for improving child health. As the contextualisation of the findings requires multidisciplinary and multisectoral stakeholder engagement, we organised a workshop in Uganda. The workshop helped build bridges between research and practice, link sectors, and connect people. We hope this workshop will become an institutionalised effort that sparks action for sustainable preventive integrated child health care and sustainable health for all.
